# Fully Transparent and Sensitivity-Programmable Amorphous Indium-Gallium-Zinc-Oxide Thin-Film Transistor-Based Biosensor Platforms with Resistive Switching Memories

**DOI:** 10.3390/s21134435

**Published:** 2021-06-28

**Authors:** Hyeong-Un Jeon, Won-Ju Cho

**Affiliations:** Department of Electronic Materials Engineering, Kwangwoon University, 20 Gwangun-ro, Nowon-gu, Seoul 01897, Korea; wjsguddns9154@kw.ac.kr

**Keywords:** amorphous oxide semiconductor, ion-sensitive field-effect transistor, resistive coupling effect, embedded resistive switching memories, multi-level state

## Abstract

This paper presents a fully transparent and sensitivity-programmable biosensor based on an amorphous-indium-gallium-zinc-oxide (*a*-IGZO) thin-film transistor (TFT) with embedded resistive switching memories (ReRAMs). The sensor comprises a control gate (CG) and a sensing gate (SG), each with a resistive switching (RS) memory connected, and a floating gate (FG) that modulates the channel conductance of the *a*-IGZO TFT. The resistive coupling between the RS memories connected to the CG and SG produces sensitivity properties that considerably exceed the limit of conventional ion-sensitive field-effect transistor (ISFET)-based sensors. The resistances of the embedded RS memories were determined by applying a voltage to the CG–FG and SG–FG structures independently and adjusting the compliance current. Sensors constructed using RS memories with different resistance ratios yielded a pH sensitivity of 50.5 mV/pH (*R*_CG_:*R*_SG_ = 1:1), 105.2 mV/pH (*R*_CG_:*R*_SG_ = 2:1), and 161.9 mV/pH (*R*_CG_:*R*_SG_ = 3:1). Moreover, when the *R*_CG_:*R*_SG_ = 3:1, the hysteresis voltage width (*V*_H_) and drift rate were 54.4 mV and 32.9 mV/h, respectively. As the increases in *V*_H_ and drift rate are lower than the amplified sensitivity, the sensor performs capably. The proposed device is viable as a versatile sensing device capable of detecting various substances, such as cells, antigens, DNA, and gases.

## 1. Introduction

In response to the increasing interest in personal health and the demand for smart healthcare technology, extensive research is being conducted on sensors that can detect hygiene conditions, diseases, biological signals, and water quality directly [1,2]. In particular, field-effect transistor (FET)-based sensors offer advantageous properties such as low feature size, high integration density, short response time, and accurate sensing operation [2,3,4,5]. However, typical ion-sensitive FETs (ISFETs) based on bulk silicon substrates are confined by the Nernst limit (a theoretical sensitivity limit of 59.14 mV/pH), which impedes their real-life application and commercialization [6,7,8]. As an alternative, silicon-on-insulator (SOI)-based dual-gate ISFETs are attracting attention owing to their ability to overcome the Nernst limit via the capacitive coupling effect between the upper and lower gate oxides of the channel layer. Nevertheless, the commercial development of dual-gate ISFETs on SOI substrates faces several challenges, such as complexity involving the fabrication of multiple capacitors, expensive substrate materials, and limitations regarding the physical properties of silicon. Recently, our group proposed a sensitivity amplification technique based on the resistive coupling effect to counteract this restriction [9]. Compared to the capacitive coupling effect, resistive coupling is freed from the intrinsic restrictions of the SOI substrate material by constructing coplanar resistors, which are cost effective and reduce the processing time. In our previous report, we used coplanar-type resistors with fixed resistances, meaning that sensitivity amplification was determined via pattern design. As the sensitivity of ISFETs depends mainly on the ion concentration of the detection target and the chemical sensitivity of its surface, flexible sensitivity amplification is desirable to increase application diversity [10,11,12,13,14,15]. In this study, we propose a fully transparent and sensitivity-programmable biosensor based on an amorphous indium-gallium-zinc-oxide (*a*-IGZO) thin-film transistor (TFT) and embedded resistive switching memory (ReRAM). To ensure the cost effectiveness and full transparency of the transducers, the *a*-IGZO TFTs were fabricated on glass substrates. The fundamental TFT structure comprises a triple gate consisting of a control gate (CG), sensing gate (SG), and floating gate (FG) to provide high sensitivity through resistive coupling. In addition, as a variable resistor for sensitivity adjustment, we applied ReRAM comprising metal–insulator–metal (ITO/Ta_2_O_5_/ITO) structures that provide non-volatile multi-level cell (MLC) properties. The resistive switching (RS) memory is embedded between the CG and FG (CG–FG) and between the SG and FG (SG–FG), and the CG and SG resistances (*R*_CG_, *R*_SG_) can be adjusted independently using set and reset operations. Furthermore, we introduced a separate extended gate (EG) sensing unit to prevent damage caused by the direct exposure of the metal gate of the transducer to pH buffer solutions. This configuration improved the reliability and stability of the transducer, thereby enabling the easy replacement of damaged sensing membranes and guaranteeing their cost effectiveness. To evaluate the performance of the transducer components, we measured the RS characteristics and resistance distribution of the ITO/Ta_2_O_5_/ITO RS memory and the electrical characteristics of the *a*-IGZO TFT. After programming the resistances of the built-in RS memories, the pH sensitivity was measured according to the resistive coupling ratio of *R*_CG_ and *R*_SG_, resulting in a high sensitivity beyond the Nernst limit. In addition, to verify the long-term stability and reliability, non-ideal behaviors such as hysteresis and drift effects were evaluated.

## 2. Materials and Methods

### 2.1. Fabrication of Transducer and Sensing Units for Sensitivity-Programmable Biosensor

Fully transparent *a*-IGZO TFTs with embedded RS memories based on a metal–insulator–metal (ITO/Ta_2_O_5_/ITO) structure were fabricated on 1 × 1 cm glass substrates (Corning Inc., New York, USA). After performing the standard RCA chemical cleaning process, a bottom FG electrode consisting of a 150-nm-thick ITO conductive film was deposited on a glass substrate using an RF magnetron sputtering system and defined using photolithography and lift-off processes. Subsequently, 60-nm-thick Ta_2_O_5_ and 60-nm-thick SiO_2_ films were deposited sequentially using RF magnetron sputtering to produce the FG insulator. For the channel layer, a 50-nm-thick *a*-IGZO film was deposited using a sputter target comprising In_2_O_3_, Ga_2_O_3_, and ZnO in a ratio of 4:2:4.1 mol%. Elemental analysis of the *a*-IGZO TFT channel was performed by X-ray photoelectron spectroscopy (XPS), in which uniform concentrations of In, Ga, Zn, and O were distributed in the depth direction, and the ratio of In:Ga:Zn was 4:2:4.1. The *a*-IGZO active channel region, which had a width of 20 μm and a length of 10 μm, was defined via photolithography and wet etching processes. Then, an ITO film with a thickness of 150 nm was deposited before the source/drain (S/D) electrode was formed using the lift-off method. Local etching of the 60 nm thick SiO_2_ film, which stacked on the 60 nm thick Ta_2_O_5_ film, was conducted via a combination of photolithography and wet etching with a buffered oxide etchant (BOE, in a ratio of 30:1) to form contact holes for the CG and SG on the FG, with the Ta_2_O_5_ film acting as an RS memory switching layer. After depositing a 150 nm thick ITO conductive film, the CG and SG were formed on the Ta_2_O_5_ RS layer via a lift-off process, thereby completing the top electrode (TE) of the embedded RS memory. Additionally, the local contact area of the bottom electrode (BE) of the RS memory was defined using photolithography to create contact holes on the SiO_2_/Ta_2_O_5_ stacked layer on the FG electrode, before being etched via reactive ion etching (RIE).

**Figure 1:**
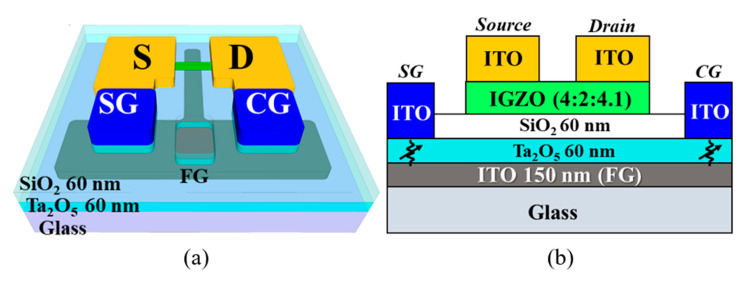
Schematic diagrams illustrating the (**a**) top-view and (**b**) conceptual cross-sectional structures of the *a*-IGZO TFT transducer with embedded ReRAMs on a glass substrate.

Finally, microwave annealing was performed at a power of 1000 W for 2 min in ambient O_2_ to improve the electrical properties of the *a*-IGZO TFT and the embedded RS memory. We performed X-ray diffraction (XRD) analysis on the as-deposited and MWA-annealed *a*-IGZO films to confirm that both were in the amorphous state. The top-view and conceptual cross-sectional structures of the *a*-IGZO TFT-based transducer with embedded ReRAMs are illustrated in Figure 1a,b, respectively. 

**Figure 2:**
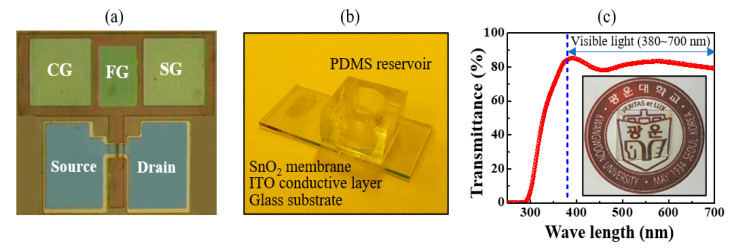
(**a**) Microscope image of the fabricated *a*-IGZO TFT transducer and (**b**) photograph of the fabricated EG. These parts were fabricated independently on separate glass substrates. (**c**) Optical transmittance and photograph (inset) of the fully transparent *a*-IGZO TFT transducer.

The EG for pH buffer solution detection was prepared by depositing a 300 nm thick ITO conductive film followed by a 50 nm thick SnO_2_ sensing membrane on a 1.5 × 2.5 cm glass substrate, and then attaching a poly-dimethyl siloxane (PDMS) reservoir to store the pH buffer solution. All the film thicknesses were measured through the DektakXT stylus profilometer (Bruker, Billerica, MA, USA) (not shown thickness data in here). Figure 2a,b show a microscope image and a photograph of the fabricated *a*-IGZO TFT transducer and the EG, respectively. Figure 2c shows the optical transmittance of the fabricated *a*-IGZO TFT transducer, which was measured over a wavelength range of 380–700 nm using an Agilent 8453 UV-visible spectrophotometer.

### 2.2. Characterization of Sensitivity-Programmable Biosensor

**Figure 3:**
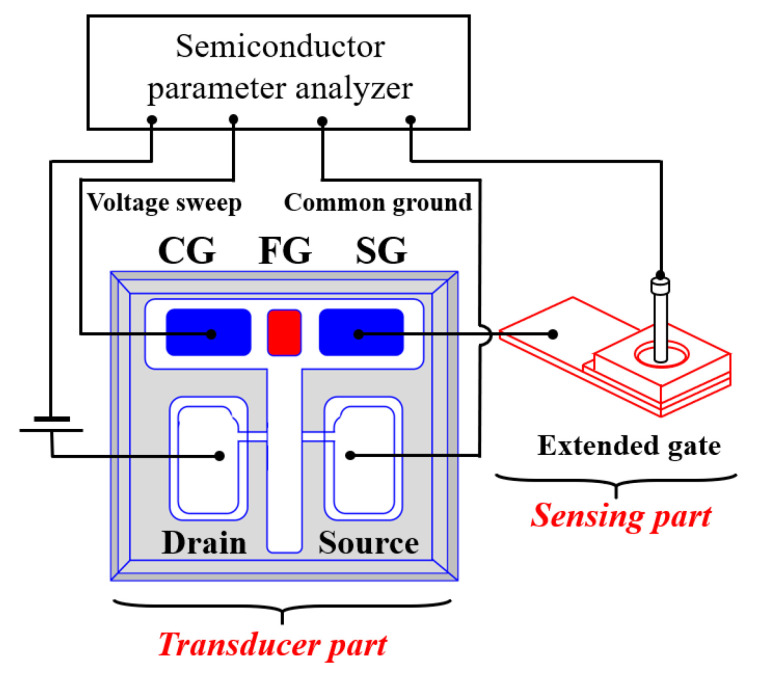
Configuration of the measurement system. The FG serves as the common BE of the embedded RS memories, while the CG and SG serve as the TE.

The electrical properties of the constructed sensors were measured using an Agilent 4156B Precision Semiconductor Parameter Analyzer and probe station system in a dark box to exclude illuminance and electrical interference. A commercial Ag/AgCl electrode (Horiba 2080A-06T) consisting of a ceramic–plug junction and an internal solution saturated with KCl and AgCl was utilized as a reference electrode for pH detection. The potential of the pH buffer solution was measured by connecting the *a*-IGZO TFT transducer and EG directly with an electric wire—the configuration of the electrical measurement system is shown in Figure 3. The sensitivity was determined by measuring the changes in the CG voltage (defined as the reference voltage, *V*_R_) at a drain current of 1 nA (defined as the reference current, *I*_R_) as a function of the pH value. In addition, the hysteresis and drift voltages were measured to identify the non-ideal behavior of the proposed sensor. The hysteresis voltage width was defined as the difference between the *V*_R_ of the first and last pH values in the following pH loop sequence: 7 → 10 → 7 → 4 → 7. To evaluate the drift phenomenon, the *V*_R_ was monitored while exposing the EG to a pH 7 buffer solution for 10 h, with the drift rate defined as the change in *V*_R_ (Δ*V*_R_). 

## 3. Results and Discussion

### 3.1. Resistive Coupling Effect Simulations

**Figure 4:**
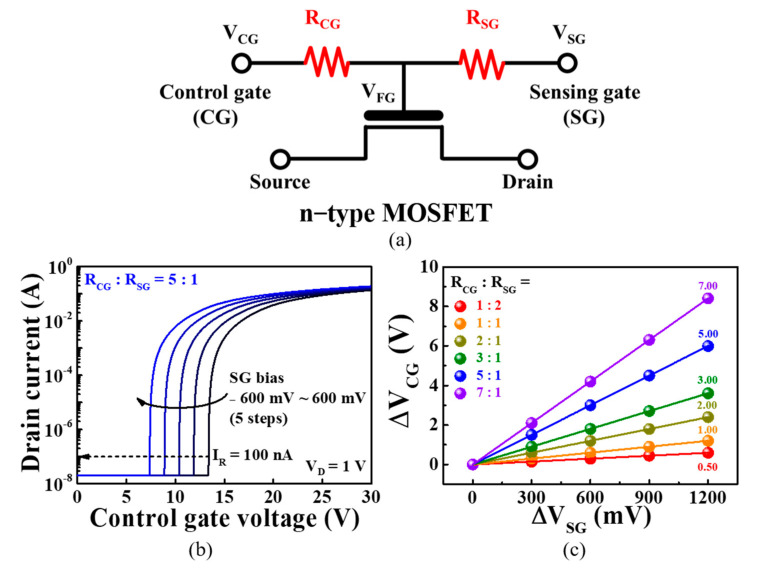
(**a**) Simplified equivalent circuit corresponding to the PSpice simulation of the sensitivity amplification due to the resistive coupling effect; (**b**) transfer characteristic curve shift as a function of *V*_SG_; (**c**) plot of Δ*V*_CG_/Δ*V*_SG_ according to the amplification factor *R*_CG_/*R*_SG_.

Figure 4 shows the simulation results (performed in PSpice) of the potential for signal amplification using resistive coupling effects, which predicts the electrical properties that may occur during practical measurements in the absence of physical and electrical interference. In addition, by excluding conduction due to unexpected tunneling phenomena, such as Fowler–Nordheim tunneling, the tunneling of conduction band electrons, trap assisted tunneling, direct quantum tunneling, and Schottky tunneling, it is possible to observe the shift of the transfer characteristics (via drain current versus gate voltage curves at a fixed drain voltage) according to the resistance ratio change with accuracy. Figure 4a shows a simplified equivalent circuit for simulating the resistive coupling effect, where *V*_CG_ and *V*_SG_ represent the voltages applied to the CG and SG, respectively, and *R*_CG_ and *R*_SG_ represent the external resistors connected in series to the CG and SG. The voltage across the FG is resistively coupled to the *R*_CG_ and *R*_SG_, as expressed by Equation (1) (in terms of voltage distribution), while the voltage across the CG is described by Equation (2). Consequently, Equation (3) can be derived, which describes the amplification of the potential modulation across the CG (Δ*V*_CG_) owing to the potential across the SG (Δ*V*_SG_) at a ratio of *R*_CG_/*R*_SG_:

(1) $$V_{\mathrm{FG}}=\left(\frac{R_{\mathrm{SG}}}{R_{\mathrm{CG}}+R_{\mathrm{SG}}}\right)V_{\mathrm{CG}}+\left(\frac{R_{\mathrm{CG}}}{R_{\mathrm{CG}}+R_{\mathrm{SG}}}\right)V_{\mathrm{SG}}\;\;$$

(2) $$V_{\mathrm{CG}}=\left(\frac{R_{\mathrm{CG}}+R_{\mathrm{SG}}}{R_{\mathrm{SG}}}\right)V_{\mathrm{FG}}-\left(\frac{R_{\mathrm{CG}}}{R_{\mathrm{SG}}}\right)V_{\mathrm{SG}}\;\;$$

(3) $$∆V_{\mathrm{CG}}\propto \left(\frac{R_{\mathrm{CG}}}{R_{\mathrm{SG}}}\right)∆V_{\mathrm{SG}}.$$

Figure 4b shows the PSpice simulation of conventional n-type MOSFET transfer characteristic (*I*_D_−*V*_G_) curves when the *R*_CG_:*R*_SG_ = 5:1 and *V*_D_ = 0.05 V, as a typical case. As the *V*_SG_ increases from −600 to 600 mV in 300 mV intervals, the transfer curve shifts in the negative direction. Figure 4c plots the magnitude of Δ*V*_CG_ as a function of *V*_SG_ for *R*_CG_:*R*_SG_ = 1:2, 1:1, 2:1, 3:1, 5:1, and 7:1. It is observed that the shift in the transfer characteristic curve increases according to the amplification factor (*R*_CG_/*R*_SG_) as *R*_CG_ increases. 

### 3.2. RS Characteristics of ITO/Ta_2_O_5_/ITO-Structured RS Memory

**Figure 5:**
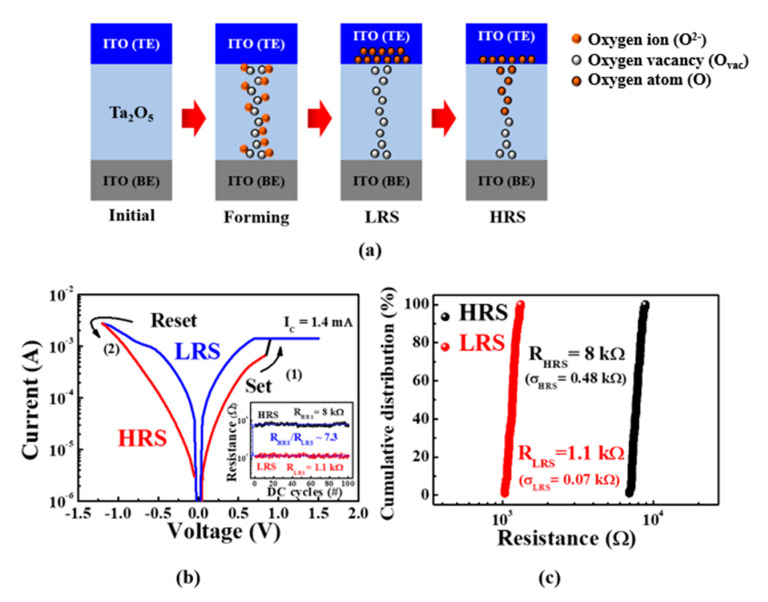
(**a**) Schematic of formation and rupturing of conductive filaments (CFs) in the Ta_2_O_5_ RS layer; (**b**) BRS *I*-V characteristics curves of ITO/Ta_2_O_5_/ITO RS layer. Inset shows resistance endurance of HRS and LRS at a read voltage (*V*_read_) of 0.2 V; (**c**) cumulative resistance distribution during 1 × 10^2^ sequential DC cycles.

Through the resistance change and non-volatile memory characteristics of the embedded RS memory of the ITO/Ta_2_O_5_/ITO structure, it is possible to decipher a continuous arbitrary resistance ratio for the fabricated sensor platform. Figure 5a exhibits the forming/rupturing process for a conducting filament (CF) in conventional metal-oxide-based RS memory. Owing to the insulating properties of the metal-oxide layer, the initial RS layer prohibits current from flowing between the TE and the BE, thus necessitating a forming process that introduces arbitrary defects into the resistance change characteristics. The forming process induces the soft breakdown of the initial RS layer by applying a strong electric field to the TE, creating oxygen vacancies (i.e., lattices in which oxygen ions are separated) and contributing to the formation of a CF through which electrons move [16,17,18,19,20]. At this point, an appropriate compliance current (*I*_C_) must be set to prevent the hard breakdown of the RS layer. Then, oxygen ions in the RS layer move according to the polarity of the electric field applied to the TE and combine with the oxygen vacancies to form and rupture CFs, with the TE acting as an “oxygen reservoir” that accumulates or releases oxygen ions. Based on this mechanism, the resistance state of the RS layer, which can be determined by the field polarity and the magnitude of the voltage applied to the TE, can be adjusted [18,20]. Figure 5b shows the typical bipolar resistive switching (BRS) *I–V* characteristics of the ITO/Ta_2_O_5_/ITO stack used in the constructed sensors, where the red and blue lines represent the high-resistance and low-resistance states (HRS and LRS), respectively. We measured the BRS characteristics at 0.01 V intervals while adjusting the voltage applied to the TE according to the following sequence: 0 V → 2 V → −1.2 V → 0 V, while setting the *I*_C_ to 1.4 mA to prevent the hard breakdown of the RS layer [16,17,18,20]. As the positive voltage applied to the TE increases, the Ta_2_O_5_ RS layer remains in an HRS until an abrupt increase in the current occurs above 0.8 V, as indicated by the “set” process labeled (1) in Figure 5b. During the “set” process, oxygen vacancies in the RS layer form CFs and the Ta_2_O_5_ RS layer becomes an LRS, resulting in a decrease in resistance, as illustrated in Figure 5a. This LRS holds irrespective of whether the voltage decreases, and the polarity is reversed; however, it changes again at a voltage of −1.2 V, whereupon the “reset” process, labeled (2) in Figure 5b, takes effect. At this point, oxygen ions stored in the TE are released into the RS layer via repulsive forces and combine with oxygen vacancies to form CFs. As a result, the CF in the RS layer ruptures and the resistance in the Ta_2_O_5_ RS layer increases, with the resistance in the RS layer “reset” to a HRS. This suggests that the fabricated ITO/Ta_2_O_5_/ITO structure can be used as an embedded variable resistor that realizes programmable sensitivity by combining resistance switching with nonvolatile memory characteristics. Inset of Figure 5b are resistance endurance of HRS and LRS at a read voltage (*V*_read_) of 0.2 V. The average *R*_HRS_/*R*_LRS_ was ~7.3, indicating that the embedded ReRAM has stable BRS endurance over 1 × 10^2^ DC cycles. Figure 5c shows the cumulative resistance distributions for LRS and HRS over 1 × 10^2^ sequential DC cycles. It is found that the embedded ReRAM with ITO/Ta_2_O_5_/ITO structure has a stable resistance distribution. The average resistances (*R*_HRS_, *R*_LRS_) and standard deviations (*σ*_HRS_, *σ*_LRS_) of the HRS and LRS were *R*_HRS_ = 8 × 10^3^ Ω (*σ*_HRS_ = 0.48 × 10^3^ Ω) and *R*_LRS_ = 1.1 × 10^3^ Ω (*σ*_LRS_ = 0.07 × 10^3^ Ω), respectively.

**Figure 6:**
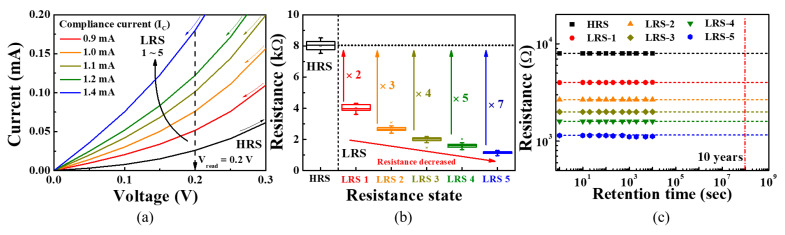
Evaluation of multi-level resistance states in ITO/Ta_2_O_5_/ITO-structured RS memory for different *I*_C_ values. (**a**) Multi-level BRS *I–V* curves at *V*_read_ = 0.2 V; (**b**) resistance distribution and (**c**) non-volatile retention performance during 10^4^ s of the multi-level resistance states.

Figure 6 shows the experimental results for the multi-level characteristics of the ITO/Ta_2_O_5_/ITO-structured RS memory. The size of the CF can be modified by adjusting the *I*_C_, which is often used to control the resistance of the RS layer. As the size of CF increases, the amount of current flowing between the TE and BE increases, thereby enabling multiple LRS levels at the same voltage [17,21,22]. Figure 6a shows the *I–V* curves as the *I*_C_ is varied from 0.9 to 1.4 mA in the “set” region where a positive voltage is applied to the TE, which is enlarged around the *V*_read_ to distinguish the resistance differences between LRS levels. Depending on the *I*_C_, a single HRS and five LRSs were identified, with the resistances of these states extracted using *V* = *IR* at the *V*_read_ of 0.2 V. To conduct a quantitative comparison and verify the uniformity of the resistance in response to repeated switching, we evaluated the RS operation 30 times for each resistance state as shown in Figure 6b. Figure 6c shows the retention characteristics for non-volatile multiple level resistance over 10^4^ s. The six resistance states do not change for 10^4^ s, providing a stable non-volatile retention characteristic. Table 1 summarizes the average resistance (*μ*) and standard deviation (*σ*) of the multi-level resistance states. As the *I*_C_ increases, the LRS resistances and their standard deviations decrease, indicating that a sensitivity-programmable sensor can be implemented using the resistive coupling of multi-level *R*_CG_ and *R*_SG_ resistors. 

### 3.3. Electrical Characteristics of a-IGZO TFT-Based Transducer Units

**Figure 7:**
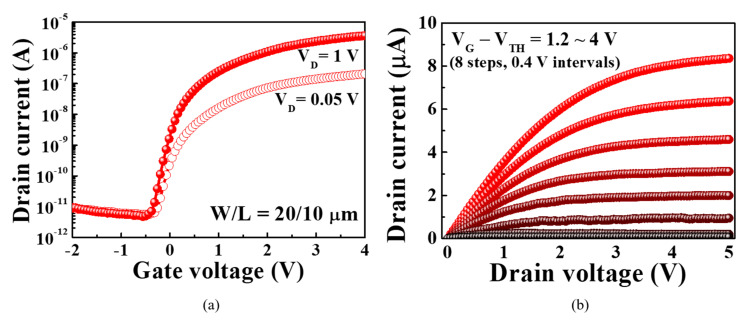
(**a**) Transfer characteristic and (**b**) output characteristic curves of the *a*-IGZO TFT-based transducer in response to the operation of the bottom FG.

Figure 7 shows the electrical characteristics of the *a*-IGZO TFT owing to the operation of the bottom FG. The performance of the transducer greatly influences the sensing operation of the entire pH sensor platform. The proposed fully transparent *a*-IGZO TFTs fabricated on glass substrates exhibit excellent electrical properties. For the transfer characteristic (*I*_D_−*V*_G_) curves in Figure 7a, the threshold voltage (*V*_th_), on/off current ratio (*I*_on_/*I*_off_), field-effect mobility (*μ*_FE_), and subthreshold swing (*SS*) were −0.44 V, 7.58 × 10^5^, 14.67 cm^2^/V·s, and 127.2 mV/dec, respectively. In addition, the interfacial trap density (*D*_it_) between the gate insulator and the *a*-IGZO channel degrades the mobility and stability of the transducer, causing non-ideal behavior during sensing. It was calculated using the following equation [23,24]:

(4) $$D_{\mathrm{it}}=\left(\frac{SSlog\left(e\right)}{kT/q}-1\right)\frac{C_{i}}{q},$$

where *q*, *k*, *T*, and *C*_i_ represent the unit charge, Boltzmann constant, absolute temperature, and capacitance per unit area, respectively. For the proposed *a*-IGZO TFT-based transducer, we determined *D*_it_ to be 3.48 × 10^11^ cm^−2^. For the output characteristic (*I*_D_−*V*_D_) curves in Figure 7b, the ohmic contact between the *a*-IGZO channel and the ITO source/drain was identified by the linear increase in *I*_D_ with *V*_D_ in the small *V*_D_ region, while a pinch-off phenomenon, whereby the *I*_D_ gradually saturates in the large *V*_D_ region, is also observed. 

### 3.4. pH-Sensing Characteristics of Sensitivity-Programmable Biosensor Platform

**Figure 8:**
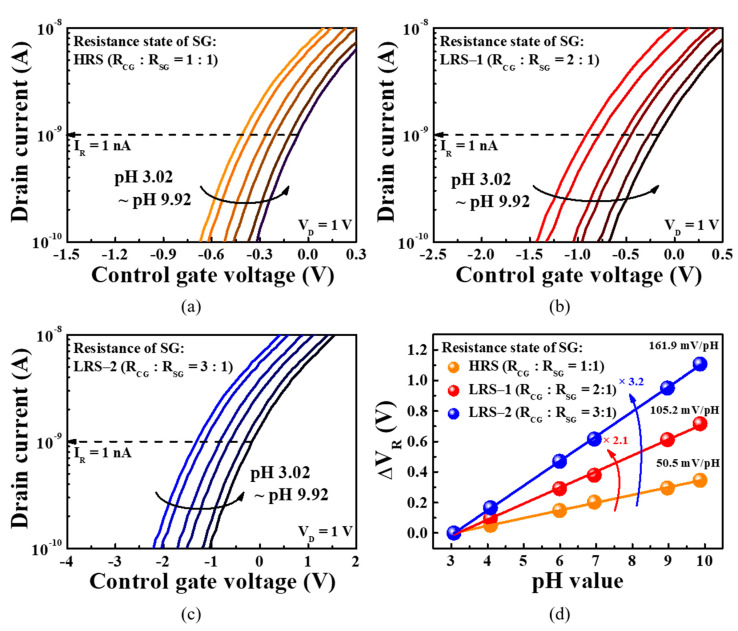
Transfer curves of the *a*-IGZO TFT transducer with ITO/Ta_2_O_5_/ITO-structured RS memory for SG resistance states of (**a**) HRS (*R*_CG_:*R*_SG_ = 1:1); (**b**) LRS-1 (*R*_CG_:*R*_SG_ = 2:1); (**c**) LRS-2 (*R*_CG_:*R*_SG_ = 3:1) in various pH buffer solutions; (**d**) pH sensitivity for various *R*_CG_:*R*_SG_ resistance ratios.

In general, the pH sensitivity at room temperature cannot exceed 59.14 mV/pH because of the Nernst limitation [6]. According to the site-binding theory, the pH-dependency of the surface potential (*ψ*) is given by

(5) $$\psi =2.303\frac{kT}{q}\frac{\beta }{\beta +1}\left(pH_{pzc}-pH\right),$$

where *pH_pzc_* is the pH value with zero free charges and *β* is the constant representing the chemical sensitivity of the sensing surface [7]. Thus, the value of *ψ* depends on the number of ions in the pH solution and the chemical sensitivity of the sensing surface. The sensitivity amplification of the proposed sensor is implemented through the following mechanisms. First, the change in the surface potential (Δ*ψ*) of the membrane in the EG unit owing to the change in the pH value of the buffer solution is transferred to the SG electrode of the transducer unit, thereby changing the *V*_SG_. Then, the *V*_CG_ (voltage across the CG electrode) connected in series with the SG electrode via the FG electrode is changed by *V*_SG_∙(*R*_CG_/*R*_SG_) owing to the resistive coupling, as described by Equation (2). As a result, the transfer characteristic curve corresponding to the *V_CG_* sweep shifts by *R*_CG_/*R*_SG_, while the reference voltage (*V*_R_) is changed by the same magnitude. Finally, the sensitivity of the proposed sensor is amplified by *R*_CG_/*R*_SG_. Figure 8a–c show the pH sensing characteristics of the sensitivity-programmable sensors. The resistance states of the *R*_SG_ were programmed as HRS, LRS-1, and LRS-2, which are chosen as typical resistance states in this study, while the *R*_CG_ remained as HRS. 

The resistance according to each resistance state is mentioned in Table 2, and when *R_SG_* is programmed as HRS, LRS-1, and LRS-2, the resistance ratio *R*_CG_:*R*_SG_ is 1:1, 2:1, and 3:1, respectively. Sensitivity measurements were performed using buffer solutions with pH concentrations of 3.02, 4.07, 5.98, 6.99, 8.98, and 9.92, and the transfer curve was obtained by sweeping the CG voltage. The magnitude of the transfer curve shift was determined using the reference drain current (*I*_R_, 1 nA), and thus the magnitude of transfer curve shift is represented by the reference voltage shift (Δ*V*_R_). Figure 8d shows the Δ*V*_R_ as a function of the pH value. For *R*_SG_ = HRS (*R*_CG_:*R*_SG_ = 1:1), the pH sensitivity was 50.5 mV/pH, which is lower than the Nernst limit of 59.14 mV/pH. However, for *R*_SG_ = LRS-1 and LRS-2 (*R*_CG_:*R*_SG_ = 2:1 and 3:1), the pH sensitivities were 105.2 and 161.9 mV/pH, respectively, exceeding the Nernst limit comfortably. Furthermore, the sensitivities according to *R*_CG/_*R*_SG_ exhibit a linearity of 99.9%. These results demonstrate that the proposed sensitivity-programmable sensor provides flexible pH detection in various situations owing to the tunability of the amplification factor.

As the sensing membrane is exposed directly to the pH buffer for a long time, surface defects caused by diverse ions give rise to non-ideal effects, which degrades the accuracy and reliability of the device [25,26,27,28,29,30]. We measured the hysteresis and drift effects to evaluate the reliability and stability of the sensitivity-programmable sensor. Hysteresis is the change in electrical properties due to the gradual reaction between the surface of the sensing membrane and the ions in the buffer solution, while the drift indicates the change in the electrical properties due to ionic penetration from the surface into the sensing membrane [25,26].

**Figure 9:**
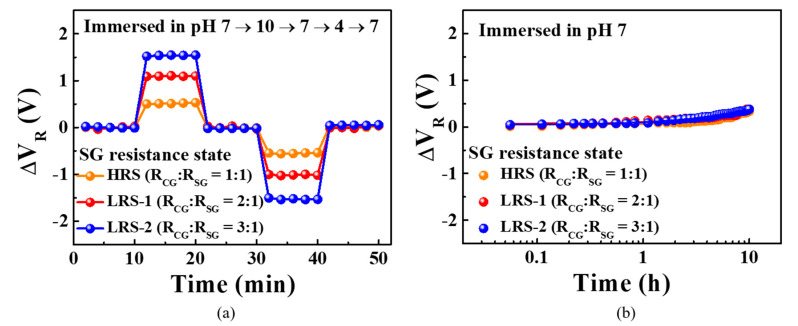
(**a**) Hysteresis and (**b**) drift effects for the sensitivity-programmable *a*-IGZO ISTFT sensor with embedded ITO/Ta_2_O_5_/ITO RS memory. Non-ideal effects were monitored for SG resistance states of HRS (*R*_CG_:*R*_SG_ = 1:1), LRS-1 (*R*_CG_:*R*_SG_ = 2:1), and LRS-2 (*R*_CG_:*R*_SG_ = 3:1).

Figure 9a shows the hysteresis effect measured by immersing the membrane in pH buffer solutions. The magnitude of the hysteresis voltage was measured as the *V*_R_ difference between the initial and final pH states of the pH loop in which the buffer solution was changed at 10 min intervals according to the following sequence: pH 7 → pH 10 → pH 7 → pH 4 → pH 7. The hysteresis voltage widths were 34.5, 45.4, and 57.4 mV for *R*_SG_ states of HRS (*R*_CG_:*R*_SG_ = 1:1), LRS-1 (*R*_CG_:*R*_SG_ = 2:1), and LRS-2 (*R*_CG_:*R*_SG_ = 3:1), respectively. Figure 9b shows the evolution of the drift effect over a 10 h period in a pH 7 buffer solution. Drift rates of 29.0, 30.4, and 32.9 mV/h were recorded for *R*_SG_ states of HRS, LRS-1, and LRS-2, respectively. Table 2 summarizes the pH sensing characteristics of the fabricated sensitivity-programmable sensor. Compared to the *R_SG_* states of HRS (*R*_CG_:*R*_SG_ = 1:1), the *R_SG_* states of LRS-1 (*R*_CG_:*R*_SG_ = 2:1) has 2.08 times higher sensitivity, 1.33 times greater V_H_, and 1.04 times higher drift rate, while when the *R_SG_* states of LRS-2 (*R*_CG_:*R*_SG_ = 3:1) has 3.21 times higher sensitivity, 1.66 times larger *V*_H_, and 1.13 times higher drift rate. Note that although the pH sensitivity of the proposed device shows a linear increase regarding the *R*_CG_:*R*_SG_ resistance ratio, non-ideal behaviors such as the *V*_H_ and the drift rate are not affected significantly by these resistance ratios, thus highlighting the potential of the fabricated device as a high-performance sensor platform.

## 4. Conclusions

In this paper, we propose a fully transparent and sensitivity-programmable biosensor platform based on an *a*-IGZO TFT with embedded ITO/Ta_2_O_5_/ITO RS memory. Using PSpice simulations, we calculated the sensitivity amplification caused by the resistive coupling of the *R*_CG_ in the CG electrode and the *R*_SG_ in the SG electrode, which were connected in series via the FG electrode. The *a*-IGZO TFT, which has a significant influence on the sensing operation, is fully transparent and fabricated on a glass substrate and exhibits excellent electrical characteristics as a transducer for the proposed sensor platform. In addition, the embedded RS memories of metal–insulator–metal (ITO/Ta_2_O_5_/ITO) structure between CG–FG and between SG–FG exhibits bipolar switching behavior, providing non-volatile multi-level cell (MLC) characteristics. The various resistances of the embedded RS memories were implemented by independently applying voltage to CG–FG and SG–FG and adjusting *I*_C_. Through this, the sensitivity of the *a*-IGZO TFT transducer with embedded ITO/Ta_2_O_5_/ITO RS memory provides various pH sensitivity according to the resistance states programmed in the RS memories embedded in the CG and SG electrodes. For programmed resistance ratios of *R*_CG_:*R*_SG_ = 1:1, 2:1, and 3:1, the pH sensitivity exhibited 50.5, 105.2, and 161.9 mV/pH, respectively, thereby demonstrating the feasibility of exceeding the Nernst limit (59.14 mV/pH). In addition, to verify non-ideal effects, we evaluated the hysteresis and drift effects, revealing that the increases in the hysteresis voltage width and drift rate are lower than the sensitivity amplification, confirming the suitability of the proposed sensor platform for high-performance sensor applications. In conclusion, the proposed platform displays immense promise for applications involving the detection of diverse substances containing different amounts of ions such as cells, antigens, antibodies, DNA, and gases or for self-calibrating differences in chemical sensitivity.

## Figures and Tables

**Table 1:** Average resistance and standard deviation (*σ*) of the resistance states in ITO/Ta_2_O_5_/ITO-structured RS memory.

Resistance State	*R* (kΩ)	*σ* (kΩ)
HRS	7.99	0.30
LRS-1 (*I*_C_ = 0.9 mA)	3.99	0.19
LRS-2 (*I*_C_ = 1.0 mA)	2.63	0.14
LRS-3 (*I*_C_ = 1.1 mA)	2.05	0.13
LRS-4 (*I*_C_ = 1.2 mA)	1.62	0.13
LRS-5 (*I*_C_ = 1.4 mA)	1.14	0.08

**Table 2:** Summary of the pH sensing characteristics of the fabricated sensitivity-programmable sensor.

Resistance State of *R*_SG_	Sensitivity (mV/pH)		*V*_H_ (mV)		Drift Rate (mV/h)	
	Measured	Amplified	Measured	Amplified	Measured	Amplified
HRS	50.5	1	34.5	1	29.0	1
LRS-1 (*R*_CG_:*R*_SG_ = 2:1)	105.2	2.08	45.4	1.31	30.4	1.04
LRS-2 (*R*_CG_:*R*_SG_ = 3:1)	161.9	3.21	57.4	1.66	32.9	1.13

## Data Availability

The data presented in this study are available from the corresponding author upon reasonable request.
